# Discovering functional linkages and uncharacterized cellular pathways using phylogenetic profile comparisons: a comprehensive assessment

**DOI:** 10.1186/1471-2105-8-173

**Published:** 2007-05-23

**Authors:** Raja Jothi, Teresa M Przytycka, L Aravind

**Affiliations:** 1 National Center for Biotechnology Information, National Library of Medicine, National Institutes of Health, Bethesda, MD 20894, USA

## Abstract

**Background:**

A widely-used approach for discovering functional and physical interactions among proteins involves phylogenetic profile comparisons (PPCs). Here, proteins with similar profiles are inferred to be functionally related under the assumption that proteins involved in the same metabolic pathway or cellular system are likely to have been co-inherited during evolution.

**Results:**

Our experimentation with *E. coli *and yeast proteins with 16 different carefully composed reference sets of genomes revealed that the phyletic patterns of proteins in prokaryotes alone could be adequate enough to make reasonably accurate functional linkage predictions. A slight improvement in performance is observed on adding few eukaryotes into the reference set, but a noticeable drop-off in performance is observed with increased number of eukaryotes. Inclusion of most parasitic, pathogenic or vertebrate genomes and multiple strains of the same species into the reference set do not necessarily contribute to an improved sensitivity or accuracy. Interestingly, we also found that evolutionary histories of individual pathways have a significant affect on the performance of the PPC approach with respect to a particular reference set. For example, to accurately predict functional links in carbohydrate or lipid metabolism, a reference set solely composed of prokaryotic (or bacterial) genomes performed among the best compared to one composed of genomes from all three super-kingdoms; this is in contrast to predicting functional links in translation for which a reference set composed of prokaryotic (or bacterial) genomes performed the worst. We also demonstrate that the widely used random null model to quantify the statistical significance of profile similarity is incomplete, which could result in an increased number of false-positives.

**Conclusion:**

Contrary to previous proposals, it is not merely the number of genomes but a careful selection of informative genomes in the reference set that influences the prediction accuracy of the PPC approach. We note that the predictive power of the PPC approach, especially in eukaryotes, is heavily influenced by the primary endosymbiosis and subsequent bacterial contributions. The over-representation of parasitic unicellular eukaryotes and vertebrates additionally make eukaryotes less useful in the reference sets. Reference sets composed of highly non-redundant set of genomes from all three super-kingdoms fare better with pathways showing considerable vertical inheritance and strong conservation (e.g. translation apparatus), while reference sets solely composed of prokaryotic genomes fare better for more variable pathways like carbohydrate metabolism. Differential performance of the PPC approach on various pathways, and a weak positive correlation between functional and profile similarities suggest that caution should be exercised while interpreting functional linkages inferred from genome-wide large-scale profile comparisons using a single reference set.

## Background

Recent advances in whole-genome sequencing have generated an avalanche of genomic sequences from diverse species. Major challenges in the post-genomic era include discovering the function of proteins, and determining how proteins interact with each other (either physically or functionally) in the context of cellular pathways and network modules. Various high-throughput experimental methods [1-9] have been used to infer functional linkages and biological interactions among proteins. In addition, several computational approaches for predicting protein functions and interactions (such as gene fusion[10,11], gene neighbors [12-14], gene co-occurrence [15-18], sequence co-evolution [19-28], co-evolution of gene expression [29,30], and correlated evolutionary events [31,32]) have been proposed in an effort to complement experimental methods.

Among computational methods available for discovering functional linkages, one simplest yet elegant approach involves phylogenetic profiles comparisons (PPCs) [17,18]. In this approach, patterns of presence or absence of protein families across multiple genomes are used to infer functional linkages between proteins. The phylogenetic profile of a protein is a vector of length *n*, which contains the presence or absence information of homologs of that protein (represented as ones and zeroes, respectively) in *n *different genomes of interest. Proteins having matching or similar profiles are inferred to be functionally linked [33-70] under the assumption that proteins involved in the same pathway or functional system are likely to have been co-inherited during evolution. PPCs allow us to predict the function of uncharacterized proteins [37,71] by simply relating profiles of proteins with known function to those of proteins whose function is unknown. Moreover, clustering protein profiles based on their similarity enables us to discover uncharacterized cellular pathways and functional network modules [71-80], and sub-cellular locations of proteins [81].

In this work, we perform a comprehensive assessment of functional linkage inference using PPCs. Our goal is to measure the approach's accuracy and coverage as well as to identify its biases, strengths and weaknesses. We studied *E. coli *and *S. cerevisiae *(yeast) proteins through comparative analysis of 894,522 known proteins from 95 different organisms. Sun et al [82] had previously reported that the choice of the reference set of genomes (genomes in which a given protein is profiled) affects predictive power of the PPC approach, albeit with no clear explanation in the evolutionary context. Results from our experiments using 16 different carefully composed reference sets of genomes show that the extent of enrichment of functionally related protein pairs among those with high similarity score is dependent on the evolutionary history of individual pathways to which the proteins belong, and thus the choice of genomes included in the reference set. For example, to accurately predict functional links in carbohydrate or lipid metabolism, a reference set solely composed of prokaryotic (or bacterial) genomes performed among the best compared to a reference set composed of genomes from all three super-kingdoms; this is in contrast to predicting functional links in translation for which a reference set composed of prokaryotic (or bacterial) genomes performed the worst.

We show that selection of genomes for the reference set both at the super-kingdom level as well as within the eukaryotic kingdom affects the predictive power of the PPC approach. In particular, our results on the *E. coli *and yeast proteins reveal that profiling evolutionary traits of protein families in prokaryotic genomes alone could be adequate to infer reasonably accurate functional linkages between proteins. Adding a few eukaryotic genomes (serving as an out-group) into the reference set results in an improved performance. However, adding too many eukaryotes into the reference set decreases the performance. We provide evolutionary explanations for the observed trend, and propose simple guidelines for the selection of reference set of genomes for phylogenetic profile analysis.

We also show that the widely used random null hypothesis, which involves comparison of the performances of the actual and the shuffled profiles, to assess the statistical significance of profile similarity scores in genome-wide large-scale functional linkage predictions could lead to a significant number of unrelated proteins pairs being predicted as functionally related. In particular, we show that using distribution of similarity scores of shuffled profiles to model functionally unrelated proteins pairs could result in a large fraction of predicted functional linkages being false-positives. In other words, we show that using shuffled profiles to evaluate the tendency of protein profiles to be similar by chance underestimates the probability of an unrelated protein pair having a certain similarity score, thereby increasing the chances of predicting false functional linkages.

## Results and discussion

**Figure 1:**
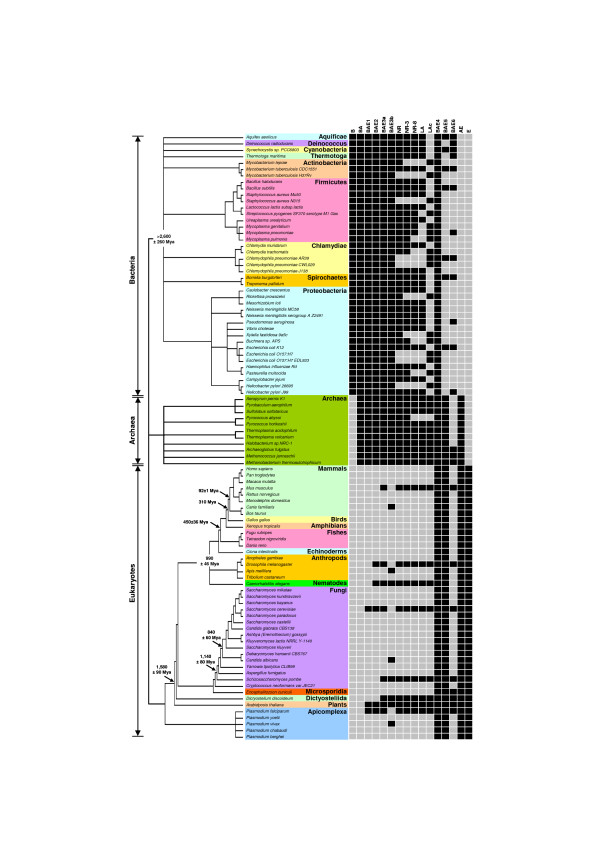
Reference sets of organisms. The relationships and divergence times (Mya – million years ago) of 95 organisms (41 bacterial, 11 archaeal, and 43 eukaryotic genomes) are based on recent studies [95]. The tree branch lengths are not proportional to time.

Phylogenetic profiles of all *E. coli *and yeast proteins were constructed by searching for their homologs in 95 different diverse organisms representing all three super-kingdoms of life (41 Bacteria, 11 Archaea, and 43 Eukaryotes). Sixteen different sets of reference genomes from 95 organisms (Figure 1 and Table 1) were carefully chosen to investigate the impact of reference set of genomes on functional linkage prediction capabilities. The Basic Local Alignment Search Tool (BLAST) [83] from NCBI was used to compare the protein sequences against each other. Every protein *i *is searched against the set of proteins from each organism *j*, and the presence/absence of the query protein's homolog in organism *j *is recorded in the form of BLAST e-value *E*_*ij*_. Phylogenetic profiles were then constructed as follows: for each protein *i*, a vector *P *was generated with each entry *P*_*ij *_= -1/log*E*_*ij *_in the vector corresponding to presence/absence information of *i*'s homolog in organism *j*. To avoid logarithm-induced artifacts, values of *P*_*ij *_> 1 are truncated to 1 [64,65,71,72]. Although, earlier works on phylogenetic profile analysis have used binary values (1/0) to record presence/absence of a protein in a given organism [18,32,33,78,84-87], using real values (0.0–1.0), as defined here, provides for different levels of sequence divergence [11,38,72,81].

**Table 1:** Summary of genomes in the reference sets

	**Bacteria**						**Eukaryotes**					
				
**Profile Architecture**	**Actino**	**Firmicutes**	**Spirochaetes**	**Proteo**	**Others**	**Archaea**	**Metazoans**	**Fungi**	**Plants**	**Apicomplexa**	**Others**	**Number of Genomes**
B	3	10	2	17	9	0	0	0	0	0	0	41
BA	3	10	2	17	9	11	0	0	0	0	0	52
BAE1	3	10	2	17	9	11	0	1	1	1	0	55
BAE2	3	10	2	17	9	11	2	1	1	1	0	57
BAE3a	3	10	2	17	9	11	3	2	1	1	1	60
BAE3b	3	10	2	17	9	11	3	2	1	1	1	60
NR	2	9	2	14	7	11	3	2	1	1	1	53
NR-3	1	9	2	12	7	11	3	2	1	1	1	50
NR-8	1	8	2	10	6	10	3	2	1	1	1	45
LA	1	5	2	10	6	10	3	2	1	1	1	42
LAc	2	5	0	7	3	10	3	2	1	1	1	35
BAE4	3	10	2	17	9	11	19	16	1	5	2	95
BAE5	1	1	1	1	3	11	19	16	1	5	2	61
BAE6	1	2	1	3	5	3	2	2	1	1	2	23
AE	0	0	0	0	0	11	19	16	1	5	2	54
E	0	0	0	0	0	0	19	16	1	5	2	43

Notes: B – All 41 bacterial genomes; BA – 41 bacterial and 11 archaeal genomes; BAE1 – BA genomes plus 3 eukaryotic genomes (*S. cerevisiae*, *A. thaliana*, *P. falciparum*); BAE2 – BA genomes plus 5 eukaryotic genomes (*D. melanogaster*, *C. elegans*, *S. cerevisiae*, *A. thaliana*, and *P. falciparum*); BAE3a – BA genomes plus 8 eukaryotic genomes (*M. musculus*, *D. melanogaster*, *C. elegans*, *S. cerevisiae*, *S. pombe*, *D. discoideum*, *A. thaliana*, and *P. falcifarum*); BAE3b – BA genomes plus 8 eukaryotic genomes (*C. familiaris*, A. *mellifera*, *C. elegans*, *C. albicans*, *S. pombe*, *D. discoideum*, *A. thaliana*, and *P. vivax*); NR – BAE3a minus 7 bacterial strains; NR-3 – NR minus 3 bacterial genomes (*M. leprae*, *R. prowazekii*, and *X. fastidiosa*); NR-8 – NR-3 minus 4 bacterial genomes (*M. pulmonis*, *C. trachomatis*, *Buchnera sp. APS*, and *P. multocida*) and 1 archaeal genome (*P. abyssi*); LA – NR-8 minus 3 bacterial genomes (*S. pyogenes SF370*, *M. genitalium*, and *M. pneumoniae*); LAc – Set of eukaryotic and archaeal genomes used in LA along with the set of bacterial genomes not included (complementary set) in LA; BAE4 – All 95 genomes under consideration (41 bacteria, 11 archaea, and 43 eukaryotes); BAE5 – All Archaeal and eukaryotic genomes plus a selected set of 7 bacterial genomes; BAE6 – A selected of 23 genomes (12 bacteria, 3 Archaea, and 8 eukaryotes); AE – All Archaeal and eukaryotic genomes; E – All 43 eukaryotic genomes.

We compared phylogenetic profiles of all pairs of 1347 *E. coli *and 1191 yeast proteins, whose functions and pathway affiliations are recorded in the KEGG pathway database [88]. We decided to use KEGG pathway maps as our gold standard set of functional linkages for the following two reasons: (i) to enable fair comparison of our results with those from Date et al [72], Sun et al [82], and Snitkin et al [89], who used KEGG pathway affiliations in their analyses, and (ii) the fact that KEGG is one of the most widely used database for verifying large-scale functional linkage predictions [42,72,82,89]. A detailed summary of the dataset used in this study is given in Table 2. The degree of similarity between two profiles was assessed by measuring the mutual information score between the profiles. The higher the mutual information score, the higher the profile similarity. Using mutual information to assess the correlation between profiles constructed using BLAST E-values is a standard practice and a well established procedure [43,64,65,72,82,89,90]. Since the relationship between profile entries is nonlinear, application of a normal correlation (such as Pearson's) to compute the profile similarity is not appropriate as it assumes that the profile entries are linearly related. For a detailed report on the use of various metrics to measure the profile similarity, we refer the reader to Glazko and Mushegian's study on phylogenetic profiles [74].

**Table 2:** Description of the dataset.

	Number of Proteins (in KEGG)		Number of possible functional linkages examined		Number of positives	
			
Pathways	*E. coli*	Yeast	*E. coli*	Yeast	*E. coli*	Yeast
All Pathways	1,347	1,191	708,645	635,628	43,968	35,674
Carbohydrate metabolism	292	241	33,153	26,565	5,450	4,859
Energy metabolism	135	134	8,128	8,001	2,113	3,060
Lipid metabolism	83	100	2,080	4,656	602	1,232
Nucleotide metabolism	108	113	5,050	5,995	3,686	5,082
Amino acid metabolism	237	216	20,100	19,900	3,328	3,620
Metabolism of cofactors and vitamins	146	113	7,875	5,253	1,085	740
Translation	80	185	3,081	15,400	1,761	10,792
Membrane transport	259		24,531		16,834	
Folding, sorting, and degradation		82		2,926		809

### General observations

**Figure 2:**
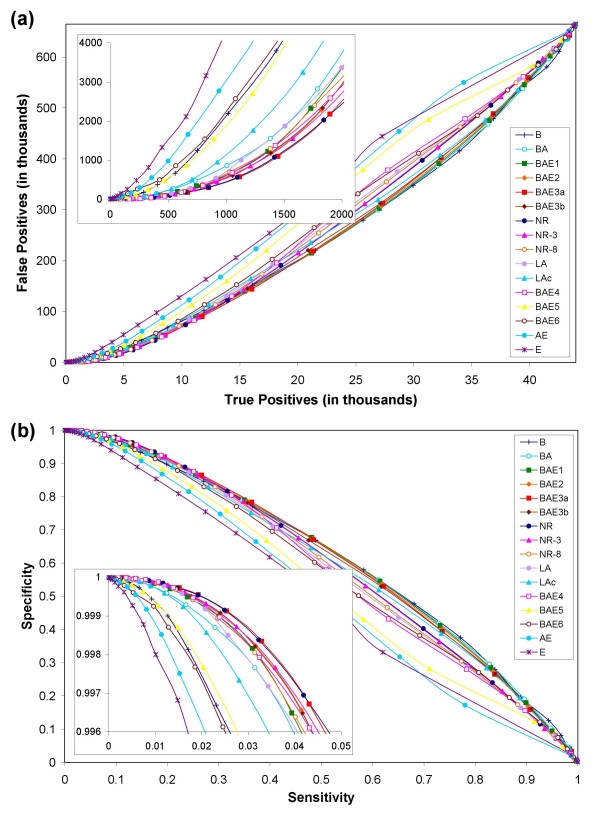
Results from phylogenetic profile comparison of 708,645 pairs of proteins chosen from among a subset of 1,347 *E. coli *proteins. (a) Predictive power of pyholgenetic profile analysis. Each point in this plot represents a specific mutual information threshold at which the measures were recorded. Reference sets with diverse bacterial genomes along with a few archaeal and/or eukaryotic genomes (BA, BAE1, BAE2, BAE3a, BAE3b, NR, NR-3, NR-8, LA, and BAE4) perform well over a reference set (B), which comprises just the bacterial genomes. The performances of BAE3a and NR are almost the same in the zoomed-in high specificity region (inset), which suggests that adding redundancy (different strains of the same organism) to the reference set does not improve the performance. The removal of evolutionarily closely-related (uninformative) genomes from the best performing BAE3a (NR-3, NR-8) decreases the performance, but to a small extent. (b) Sensitivity versus specificity plot.

**Figure 3:**
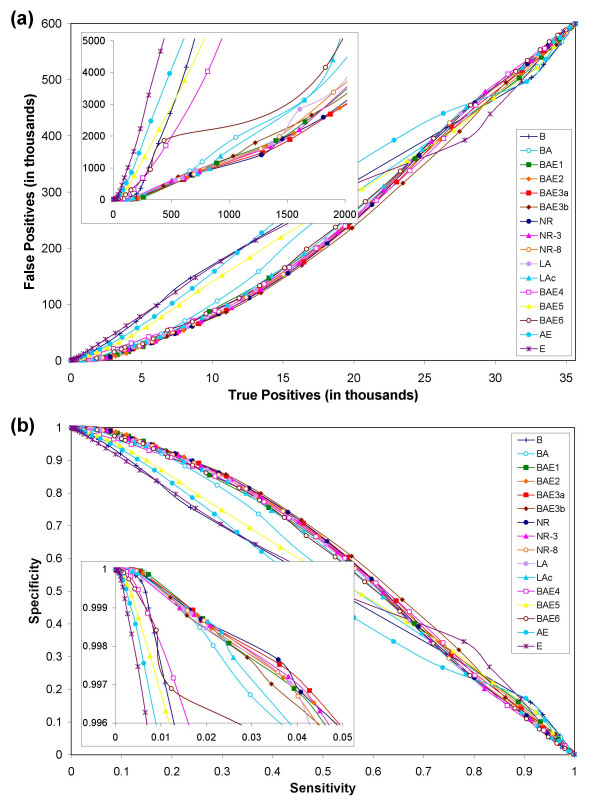
Results from phylogenetic profile comparison of 635,628 pairs of proteins chosen from among a subset of 1,191 yeast proteins. Plot representations are same as in Figure 3. (a) Predictive power of pyholgenetic profile analysis. The relative performances of all but one (BAE4) reference sets of genomes for yeast are almost the same as that for *E. coli*. A gradual decrease in performance can be noticed as the fraction of eukaryotic genomes in the reference set increases (BAE4, BAE5, BAE6, AE, E), which is counter-intuitive considering the fact that one would hope to see an improved performance on adding more eukaryotic genomes. This suggests that the diverse physiology of eukaryotes could be adding substantial noise to the protein profiles making it difficult for the method to separate functionally related protein pairs from unrelated protein pairs. Use of bacterial and archaeal genomes (BA) alone results in a good performance, a result that lends support to serial endosymbiosis theory. (b) Sensitivity versus specificity plot.

We examined a total of 708,645 possible functional linkages in *E. coli*, and 635,628 possible functional linkages in yeast using each of the 16 different reference sets of genomes. We considered two proteins to be functionally related (or linked) if they co-occur in at least one KEGG pathway [42,72]. Two proteins are inferred to be functionally related if their mutual information score is above a certain threshold. For each of 16 reference sets of genomes, performance measures for various mutual information thresholds were recorded. The overall performances using all 16 reference sets of genomes are depicted in Figures 2 and 3 for *E. coli *and yeast proteins, respectively.

**Figure 4:**
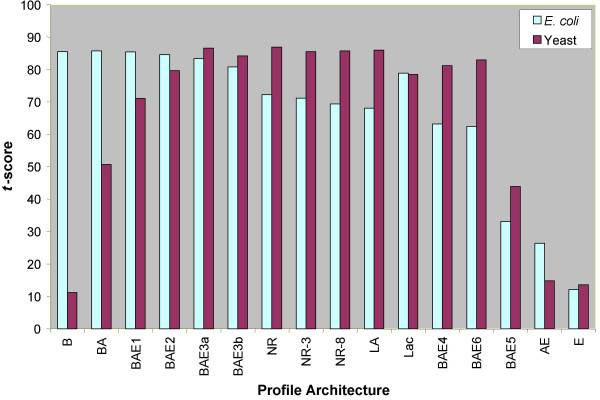
Results from the *t*-tests measuring the statistical significance of the difference in the means of the distributions of mutual information scores of random protein pairs and protein pairs with pathway similarity ≥ 50%. The t-scores for all 16 reference sets of genomes are statistically significant for both the *E. coli *and the yeast proteins.

We performed *t*-tests to determine which of the reference sets of genomes results in a statistically significant enrichment for functionally related protein pairs among all protein pairs ranked by their mutual information score. For this test, we defined pathway similarity scores (as defined in [72]) for all pairs of *E. coli *and yeast proteins, measuring the degree of functional similarity between two proteins. Pathway similarity between two proteins was computed by taking the Jaccard coefficient of their KEGG database pathway memberships (see Materials and Methods for details). Informally, two proteins *A *and *B *having a pathway similarity score of *s *indicates that *A *is present in at least *s*% of the pathways that *B *is present in, and vice-versa. For every reference set of genomes, we computed the mean mutual information score for all pairs of proteins, and pairs of proteins with ≥ 50% pathway similarity score. We then computed the *t*-score, which determines whether the difference in the two mean values is statistically significant. The *t*-score needed to be >3.29 (for risk level 0.001) in order for the difference in the mean values to be statistically significant. The test results showed that the differences in mean values for all 16 reference sets of genomes are statistically significant (Figure 4). However, the *t*-scores spanned a wide range, suggesting dramatic differences in the performance of individual reference sets.

Comparison of the results for *E. coli *and yeast proteins showed that the relative performances of all but one (BAE4) reference set of genomes were consistent (Figures 2 and 3). BAE4 did particularly poorly in yeast in the high-specificity range (insets in Figure 3) as against *E. coli *where it ranked close to the best performing reference sets (insets in Figure 2; see below for further discussion). The curves show that the ratios of false positives to true positives dramatically differ even in the high-specificity range of the curve (insets in Figures 2a and 3a). For example, for a true positive count of 500, the number of false positives ranged anywhere from a few hundreds to few thousands for different reference sets of genomes.

It is true that most curves in the sensitivity versus specificity plot (Figures 2b and 3b) seem to lie just above the diagonal. However, the zoomed-in inset (high specificity region, which represents only 0.4% of the y-axis in the bigger plot) shows that there is at least a 10-fold enrichment of correctly identifying a positive over incorrectly predicting a negative to be a positive. For example, recovering 4–5% of positives will result in incorrectly classifying 0.4% of negatives to be positives. The superior performance of PPC in the high-specificity regions of the plot (inset) is clearly evident. While PPC is a good predictor of functional linkages, especially in cases where the similarity score between functionally related profiles is very high, it may not be a great predictor. Highly accurate predictions (high specificity) using PPC can be made at the cost of low coverage (low sensitivity, insets in Fig 2 and 3). PPCs are poor tools for function prediction only if accuracy is compromised for coverage.

We investigated in detail the predictive power of the PPC approach and found that it was greatly influenced by the selection of reference set of genomes. In itself this observation is quite intuitive, and has been previously made, albeit with no clear explanation in evolutionary terms. For instance, Sun et al. [82] showed that increased number of genomes in the reference set correlates well with improved performance, and Snitkin et al [89] reported that phylogenetic profile analysis using profiles generated from the current set of completely sequenced eukaryotic organisms yields extremely poor results. Our results indicate that it is not merely the number of genomes, but a careful selection of informative genomes that is essential for an improved performance. A carefully chosen set of non-redundant genomes (NR, NR-3, NR-8, LA) results in a performance as good as if not better than the one obtained from using a larger or all-inclusive sets of genomes (BAE3a, BAE3b, BAE4; see below for further discussion).

The wide range of *t*-scores for different reference sets of genomes (Figure 4) prompted us to examine the role played by the reference set of genomes in the performance of PPCs. We also investigated the performance of different reference sets on proteins belonging to specific KEGG pathways. We present below a summary of findings emerging from these considerations.

### Selection of reference set genomes at the super-kingdom level is crucial

Using reference sets entirely composed of genomes from individual super-kingdoms, like bacteria (B) or eukarya (E), or the archaeo-eukaryotic (AE) lineage taken as a whole, results in a performance much worse than that obtained using a set that includes genomes from only the bacterial and archaeal super-kingdom (BA). The improved performance of the latter set (BA) suggests that phyletic patterns of proteins in bacterial and archaeal genomes alone provide sufficient information for reasonably accurate functional linkage predictions (also supported by statistically significant *t*-scores, Figure 4). Any further improvements to this performance necessarily require additions of a few eukaryotic genomes (e.g. BAE3a, BAE3b, NR, NR-3, NR-8, LA, LAc). The best performance was obtained for reference set BAE3a, which contained all bacterial and archaeal genomes included in our set and a single representative from each of the major eukaryotic lineages. However, further additions of eukaryotic genomes have little or no effect on the performance in *E. coli*, while they actually reduced the performance in yeast (BAE4 in Figures 2 and 3).

### Genome choice within the super-kingdoms is a notable determinant of PPC performance

There are numerous parasitic or pathogenic representatives found amongst the currently available eukaryotic and bacterial genomes. These organisms typically lack many metabolic pathways found in their sister clades, and in the extreme case are reduced to minimal genomes that mainly support only highly conserved house-keeping processes (e.g. Mycoplasmas in bacteria and *Encephalitozoon *in eukaryotes). It should also be noted that the sequencing efforts to date have been biased towards these parasitic or pathogenic organisms resulting in their over-representation in the public sequence databases. We hence decided to evaluate the effects of their inclusion (into the reference set) on the performance of the PPC approach, and observed that the inclusion or non-inclusion of such parasitic or pathogenic genomes in the reference set did not alter the performance significantly. This suggested that these genomes do not code enough proteins of various metabolic pathways to provide useful information to have a notable effect on the performance of the PPC approach. The eukaryote *Giardia lamblia *is a facultative parasite, but its genome is comparable to that of free-living unicellular eukaryotes in size and, in certain models of eukaryotic phylogeny, is considered a representative of an early branch of the eukaryotic tree. Sequence analysis of the *G. lamblia *genome also suggests a relatively robust representation of diverse functions. Interestingly, its inclusion in the best performing reference set BAE3a did not notably improve the predictive performance (not shown). This might imply that the relative time of branching of the extant eukaryotic tree does not have an influence on the predictive performance of the PPC approach.

It is also known that current databases contain numerous representatives of closely related taxa. For example, there are several representative strains of *E. coli *and numerous species of Mycoplasmas in the case of bacteria, and several *Plasmodium *and *Saccharomyces *species in the case of eukaryotes (Figure 1). Likewise, the 13 vertebrate species represented in the tree in Figure 1 do not differ significantly in most aspects of the cellular pathways considered in the KEGG database. Alternative reference sets (BAE3a, BAE3b, NR, NR-3, NR-8, LA, LAc) composed of either all the taxa or merely single representatives from each species or lineage (in the case of vertebrates) did almost as well or better than the set (BAE4) composed of all genomes (insets in Figures 2 and 3). In fact, reference set NR that contained only one representative strain for each species in bacteria did as well as the best performing BAE3a. These results indicate that different bacterial strains, closely related species, and the entire vertebrate lineage are largely redundant in terms of the information they provide for phylogenetic profile analysis.

In order to assess the possible effects arising from incomplete genome assembly and sequencing, and poor gene-predictions, we used a reference set (BAE3b) composed of poorly annotated genomes in place of their better annotated relatives. Reference set BAE3b is essentially the same as the best performing BAE3a except that we replaced *Homo sapiens *with *Canis familiaris*, *Saccharomyces cerevisiae *with *Candida albicans*, *Drosophila melanogaster *with *Apis melifera*, and *Plasmodium falciparum *with *Plasmodium vivax*. These replacements resulted in a slight drop-off in the predictive performance (insets in Figures 2 and 3), suggesting that the genomes that have been relatively not-so-well annotated vis-à-vis those well-annotated classical model organisms contain sufficient information to be of value in large-scale predictive experiments, at least for the current assembly of metabolic pathways from the KEGG database.

Another interesting effect on the performance of the PPC approach was seen specifically in eukaryotes – addition of more eukaryotes to the reference set (BAE4) did not improve the performance in the case of yeast, instead resulting in a significant drop off (Figures 3 and 4). At first sight this observation is counterintuitive, but a more careful examination of the metabolic capabilities of the genomes under consideration suggests a possible explanation for this. Most eukaryotes in this set belong to rather diverse metabolic categories, whose main "metabolic tendencies" may be very different from that of yeast. For example, the plants are photosynthetic autotrophs, animals and *Dictyostelium *are predatory deriving most nutrients by directly ingesting other eukaryotes or prokaryotes, apicomplexa are parasites deriving a greater fraction of their essential metabolites from animal hosts, whereas fungi are saprophytic or fermentative heterotrophs. As a result, there is a strong tendency for the metabolic pathways to be entirely distinct within these eukaryotes. Additionally, due to their following of different convergent paths to similar metabolic tendencies, there are widely different patchy-retentions of components of various pathways even between groups with similar overall metabolism (this is accentuated by the relative over-representation of various parasitic eukaryotes amongst the sequenced genomes). This results in distinct patterns of various protein components that might show a low degree of phylogenetic congruence. As a result, having these eukaryotic genomes together is equivalent to having a reference set in which individual proteins have been removed in an uncorrelated fashion. Thus, the addition of more such eukaryotic genomes decreases the predictive power of PPCs for functional linkage discovery in yeast (BAE4 in Figures 3 and 4).

### Structure and evolutionary history of individual pathways influence performance of PPCs

**Figure 5:**
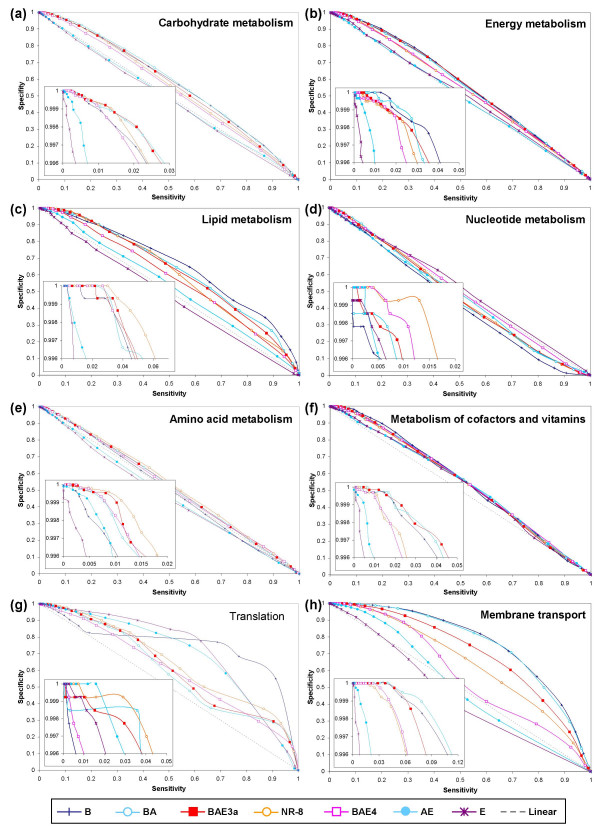
Sensitivity versus specificity plots for protein pairs in various *E. coli *pathways based on the 2nd level of KEGG orthology. Performances were measured at different mutual information thresholds. (a) Carbohydrate metabolism. (b) Energy metabolism (c) Lipid metabolism (d) Nucleotide metabolism (e) Amino acid metabolism (f) Metabolism of cofactors and vitamins (g) Translation (d) Membrane transport.

**Figure 6:**
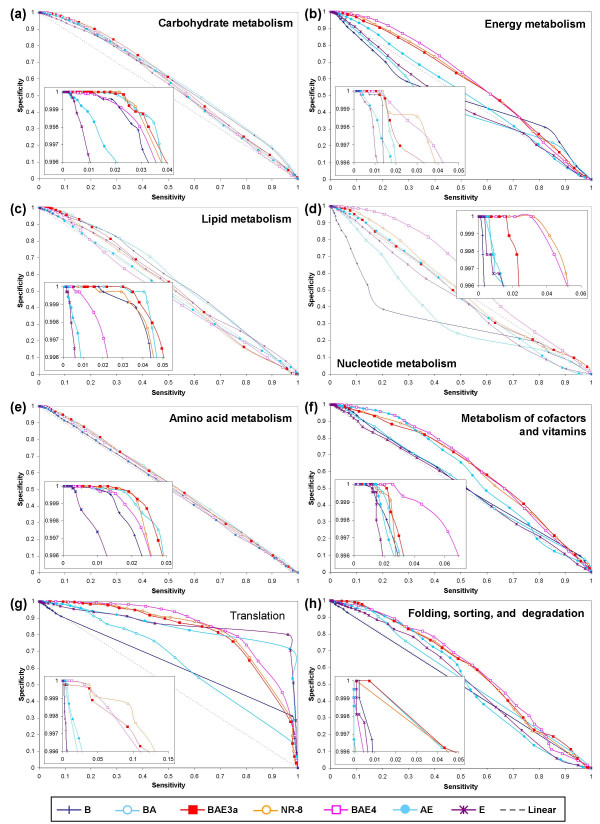
Sensitivity versus specificity plots for protein pairs in various yeast pathways based on the 2nd level of KEGG orthology. Performances were measured at different mutual information thresholds. (a) Carbohydrate metabolism. (b) Energy metabolism (c) Lipid metabolism (d) Nucleotide metabolism (e) Amino acid metabolism (f) Metabolism of cofactors and vitamins (g) Translation (h) Folding, sorting, and degradation.

A KEGG pathway or a map is an ensemble of many smaller pathways that are typically centered on a particular metabolite (e.g. DNA or RNA) or a distinct class of related molecules (e.g. carbohydrate or amino acids). It is a well-known fact that different pathways differ vastly in terms of the conservation patterns of their components. Most genome-wide large-scale functional linkage predictions using PPCs have largely ignored this intrinsic diversity in the behavior of individual biological functional systems. To evaluate the role of this diversity in conservation across different functional systems, and its effects on the accuracy of functional linkages predicted from PPCs, we considered a set of nine such systems as defined in the second level of KEGG orthology [91] (Table 2; seven are seen in both test species, while one each are found only in *E. coli *and yeast), each with 80 or more protein components. We then repeated the same analysis (as done for the complete protein set) for proteins in each of these KEGG pathways using seven of the 16 reference sets (Figures 5 and 6). For our analysis on individual pathways, we considered a pair of proteins to be a positive if they co-occur in the pathway under consideration. Otherwise, we consider the pair to be a negative (with respect to the pathway under consideration), although they may co-occur in some other pathway. This is different from our overall analysis where a pair of proteins is a positive if they co-occur in any pathway and a negative if they do not co-occur at all.

The translation apparatus is the most conserved of all pathways in the cell, with the majority of its components displaying what has been termed the "standard model" topology. In this topology, the archaeo-eukaryotic lineage is monophyletic to the exclusion of the bacteria. This phylogenetic topology is also reflected in the structure of the ribosome and principal translation switches (the GTPase complexes). Interestingly, comparisons of the prediction capabilities (sensitivity versus specificity) of the PPC approach, using various reference sets, for translation in isolation (Figures 5g and 6g) showed that neither in *E. coli *nor in yeast did the overall-best-performing reference set BAE3a fared the best. Instead, the most non-redundant reference set NR-8, comprising 15 less genomes compared to BAE3a, performed the best. The reference set composed entirely of genomes from the super-kingdom to which the test organism belonged (set B for *E. coli*, and set E for yeast) performed the worst for the translation system (Figures 5g and 6g). However, at least in the case of *E. coli*, set B fared amongst the best in 5 out of the 8 KEGG pathways (Figure 5a, b, c, f, and 5h). This observation indicated that the nature of conservation of components indeed has a notable effect on the performance of PPCs with respect to a particular reference set. In a system like translation, the phyletic pattern of a protein within a super-kingdom is rather uniform due to high degree of vertical conservation of components. Hence, profiles constructed using a reference set, composed entirely of genomes from the super-kingdom to which the test organism belongs, are likely to contain low information content and offer no predictive value. Again, due to the high degree of conservation, the phyletic pattern of a protein from a set of diverse and non-redundant genomes (NR-8) is likely to capture all the useful evolutionary information necessary for a successful functional linkage inference.

A very different picture is seen in the case of what have been described as functions associated with the "variable shell" or those functions that show great phylogenetic diversity across bacteria (e.g. carbohydrate, lipid and cofactor metabolisms). In these cases, the reference set comprised entirely of genomes from the super-kingdom to which the test organism belongs (B in the case of *E. coli*) usually performs well, though the best reference set is typically BAE3a or NR-8 (Figure 5). Given the relatively high membership of these pathways (Table 2), the dominating performance of BAE3a for the overall set of *E. coli *proteins (Figure 2) is not surprising. The more puzzling observation is the relative success of B, compared to other reference sets, in these pathways as opposed to that for the overall set. However, when the performances of various reference sets in yeast are compared, we invariably find E, and even AE, to be performing the worst for the equivalent metabolic pathways (Figure 6). Consistent with the overall results (Figure 3), like in *E. coli*, we observed that BA was close to the best even in yeast (Figure 6). Taken together, these observations suggest, that the evolutionary history of these pathways strongly affects how the proteins within them might behave as targets for function prediction using phylogenetic profiles.

Within the prokaryotes, many metabolic pathways, especially those related to carbohydrate, lipid and cofactor metabolism, are modular in structure. This feature has allowed dispersal of individual modules of the systems due to lateral gene transfer of operons, resulting in sufficiently informative phyletic patterns to provide information for predictive success (B and BA in Figures 5 and 6). There is also considerable evidence that eukaryotes acquired many of their basic metabolic abilities from bacteria, during the primary endosymbiosis (from the mitochondrial progenitor) and secondarily in some lineages due to photosynthetic endosymbiosis or via the sporadic uptake of bacteria due to phagocytosis. As a result, it is not surprising that phyletic patterns of proteins in prokaryotic genomes alone (reference sets B and BA) are sufficient to provide a high fidelity prediction of eukaryotic metabolic systems. As explained earlier, the metabolic situation of the current set of eukaryotes makes reference sets E and AE unsuitable for function prediction.

### Weak positive correlation between profile similarity and functional similarity

**Figure 7:**
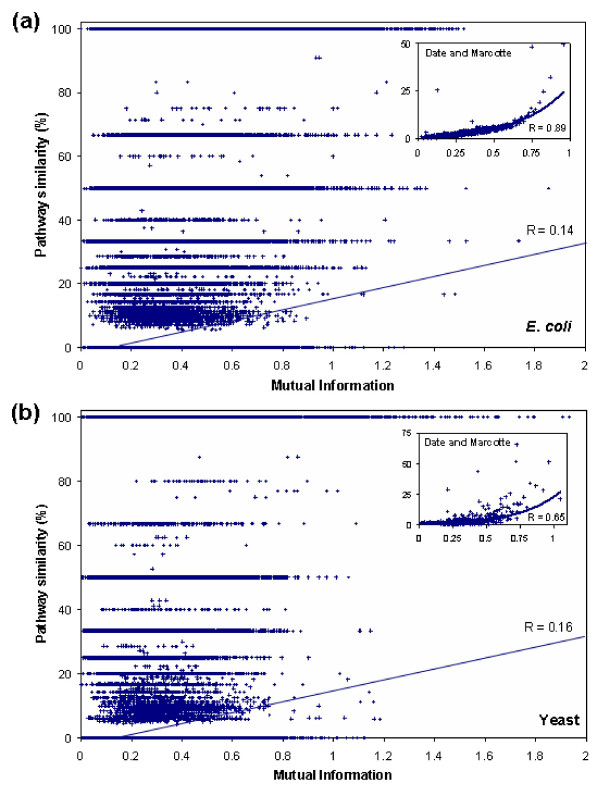
Relationship between pathaway similarity score, measured as the Jaccard coefficient between the proteins' KEGG pathway memberships, and profile similarity score (using reference set BAE3a), measured as the mutual information score of proteins' profiles. Each data point in the plot represents a pair of proteins. (a) 708, 645 pairs of *E. coli *proteins, out of which 664,677 had zero pathway similarity score. A weak positive correlation (R = 0.14) is found to exist between the pathway similarity score and the mutual information score. Rather than computing the correlation of all data points, Data and Marcotte computed the correlation of "representative" data points, each of which represents the average values for 1000 data points. This results in an artificial increase in the correlation (R = 0.89, inset). (b) 635 628 pairs of yeast proteins, out of which 599,954 had zero pathway similarity score. A weak positive correlation is observed (R = 0.16) between the pathway and profile similarity measures. An artificial increase in the correlation is observed (R = 0.65, inset) when Date and Marcotte's correlation computation strategy is employed.

Date and Marcotte [72] reported that higher mutual information scores, measuring the profile similarity, correlate well with increasing pathway similarity scores. Using exactly the same reference set of genomes and dataset, we observed that there is only a weak correlation between the two measures (0.14 foe *E. coli*, and 0.16 for yeast; Figure 7). While very high mutual information scores certainly correlate well with high pathway similarity scores, high pathway similarity scores do not, however, correlate with high mutual information scores. The main reason that Date and Marcotte observed a higher correlation than what we observe is the methodology they employed to compute the correlation. Each data point in our plot (Figure 7) represents a pair of proteins while each data point in Date and Marcotte's plot represents the average value for 1,000 pairs of proteins. They first sorted the mutual information scores, and then binned them into groups of 1000. Each bin is represented in their plot by a single data point using the bin's average mutual information score and pathway similarity score (insets in Figure 7). Consequently, rather than computing the correlation of all data points, they computed the correlation of the representative data points (averages), which resulted in an artificial increase in the correlation.

A weak positive correlation between the profile similarity score and the pathway similarity score along with substantial differences in the evolutionary modularity of functional modules [80] suggest that although PPCs have been proven to be extremely useful in inferring functional linkages on a case-by-case basis, caution should be exercised while interpreting functional linkages predicted from genome-wide large-scale PPCs using a single reference set.

### Correct formulation of null hypothesis is vital for increased prediction accuracy

In this section, we show that using an incomplete random null model can artificially boost the statistical significance of the observed functional linkages. Since choosing the correct random null model is critical to genome-wide large-scale functional linkage prediction, it is essential that one uses a complete null model that considers the underlying functional and evolutionary constraints. Our goal was not to suggest a null model that realistically captures all the issues that may arise, but merely to explore the implications of choosing an incomplete null model. Genome-wide prediction of functional linkages or cellular pathways (by clustering functional linkages) using PPCs involves selecting a profile similarity score cutoff, which is usually done by comparing the distribution of similarity scores of actual profiles to that of shuffled profiles (obtained by random shuffling of the profile entries) [72,75,77,82,92]. A similarity score cutoff is chosen such that the probability that a pair of shuffled profiles have a score greater than the cutoff is statistically very low. All protein pairs whose similarity scores (obtained from actual profiles) exceed the chosen cutoff are inferred to be functionally related. This strategy assumes that the distribution of similarity scores of shuffled profiles approximate the distribution of similarity scores of random (or unrelated) pairs of proteins.

**Figure 8:**
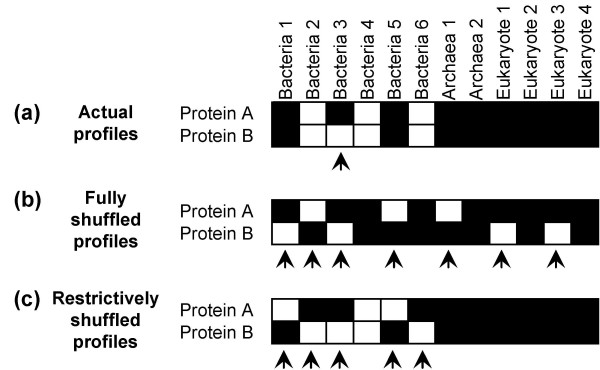
Phylogenetic profiles of two bacterial-specific proteins *A *and *B*. The presence or absence of homolog in a genome is represented by white and black squares, respectively. Bit differences in the corresponding positions of the profiles are shown using arrows. (a) The actual profiles of *A *and *B *are similar, with only one bit difference. (b) A shuffling strategy that shuffles all the entries in the profile will result in at most 7 bit differences. (c) A restrictive shuffling mechanism (which takes into account the lineage-specificity of the proteins under consideration) that shuffles only the entries corresponding to bacterial genomes will result in at most 5 bit differences. For lineage-specific proteins, unrestricted shuffling process can artificially reduce the similarity scores of the shuffled profiles, thereby underestimating the probability of a random protein pair having a certain similarity score.

The approach outlined above can lead to wrong interpretations (increase the fraction of false positives) if the underlying null hypothesis is not posed carefully. The shuffling mechanism, which shuffles all entries of a profile, implicitly assumes that the protein family, whose profile is being shuffled, is present in all the genomes under consideration. In other words, it is assumed that all proteins in the test organism date back to the last common ancestor. This type of shuffling fails to take into account those proteins that are kingdom (or lineage) specific. Consider the profiles of two bacterial-specific proteins *A *and *B *in Figure 8. Homologs of protein *A *and *B *are present in three and four bacterial genomes, respectively. Comparison of profiles *A *and *B *indicate that they are very similar, with only one bit difference (Figure 8a). The widely-used shuffling strategy could shuffle the profiles to have at most seven bit differences (Figure 8b). However, a restrictive shuffling (that takes the lineage-specificity of the proteins into consideration), which shuffles only the entries corresponding to bacterial genomes, will result in at most five bit differences (Figure 8c). This example illustrates how an unrestricted shuffling process could artificially reduce the similarity scores of the shuffled profiles, underestimating the probability of a random protein pair having a certain similarity score. Consequently, this could result in choosing a lower similarity score cutoff, thereby increasing the chances of predicting functional linkages between unrelated protein pairs. As a result of underestimating the probability of a random protein pair having a certain similarity score, the statistical significance of actual profiles having high similarity scores is erroneously overestimated.

**Figure 9:**
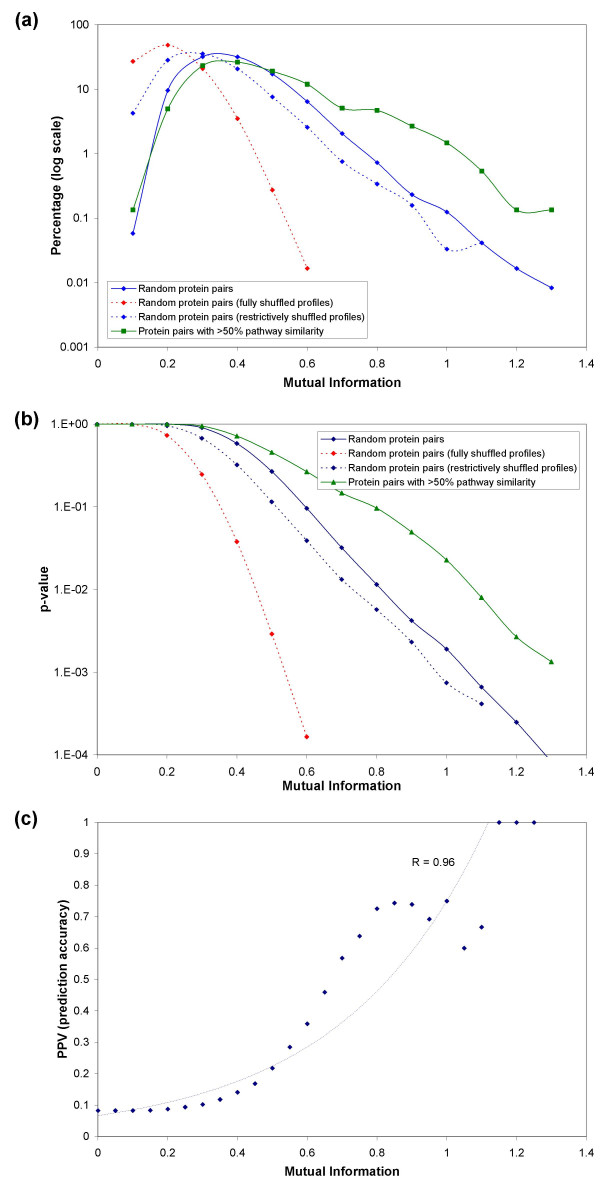
Consequences of choosing a mutual information score threshold (for predicting functional linkages among proteins) based on fully shuffled protein profiles. Results from phylogenetic profile comparison of 11,935 pairs of 155 bacterial-specific *E. coli *proteins. (a) Distribution of mutual information scores using reference set BAE4. The blue curve represents the distribution of scores for all 11,935 protein pairs (using actual profiles), while the green curve represents the distribution of scores for 983 protein pairs (using actual profiles) with ≥50% pathway similarity. The dashed curves represent the distribution of scores for 11,935 protein pairs using shuffled profiles. The dashed red curve is used for shuffled protein profiles obtained by shuffling all of the entries in actual protein profiles. This type of shuffling implicitly assumes that each protein under study is present in all lineages/kingdoms, an assumption that is incorrect for lineage-specific proteins. The dashed blue plot is for restrictively shuffled protein profiles that were obtained by shuffling only the profile entries corresponding to bacterial genomes. (b) Plots depicting the relationship between the mutual information threshold and the p-value (probability that the score for a pair of proteins meets or exceeds the chosen mutual information threshold). The statistical significance for a pair of proteins having a certain similarity score (using actual profiles) could be overestimated if fully shuffled profiles (dashed red curve) were used to model the behavior of unrelated pair of proteins instead of restrictively shuffled profiles (dashed blue curve). (c) Relationships between the mutual information thresholds (for predicting positives and negatives) and the positive predictive values (prediction accuracy). This plot illustrates that the commonly used approach of choosing a threshold based on the distribution of scores from completely shuffled profiles (0.6 based on the dashed red curve in (a)) may lead to a significant fraction of predictions being false-positives. On the contrary, a cutoff chosen based on the distribution of scores from restrictively shuffled profiles (1.1 based on the dashed blue curve in (a)) more than doubles the prediction accuracy, albeit decreasing the coverage.

To demonstrate this point further, we considered a set of 155 bacterial-specific *E. coli *proteins (see Additional File 1), whose functions and pathway affiliations are known and recorded in the KEGG pathway database. We designated an *E. coli *protein to be bacterial-specific, if a BLAST search of that protein did not fetch a homolog (with BLAST e-value < 1e-2) in any of the archaeal or eukaryotic genomes. Using reference set BAE4 (comprising all 95 organisms), we computed the mutual information scores of 11,935 pairs of 155 proteins. The distribution of mutual information scores is shown in Figure 9a. While the distribution of scores for fully shuffled profiles show that the probability of obtaining a score > 0.6 is less than 2 × 10^-4 ^(dashed red curve in Figure 9b), the distribution of scores for restrictively shuffled (only bacterial entries were shuffled) profiles show that the probability of exceeding a score of 0.6 is actually less than 2 × 10^-2 ^(dashed blue curve in Figure 9b). Using fully shuffled profiles to benchmark a mutual information cutoff for functional linkage prediction would have underestimated the probability of a random protein pair having a score of 0.6 by two orders of magnitude. If one were to choose a cutoff of 0.6 to predict functional linkages based on an incomplete random model (shuffling all the entries in a profile), majority (≈65%) of the predictions would have turned out to be false-positives (Figure 9c). However, a cutoff (1.1) chosen based on the distribution of partially shuffled profiles would have reduced the percentage of false-positives by half to about 35%.

In addition to emphasizing the importance of choosing a correct random model, the above discussed case also brings to light the limitations of the PPC approach when it comes to lineage-specific proteins. Especially, when the proteins under study are observed to occur in only a few closely-related genomes (say, in gamma-proteobacteria) the profiles of these proteins becomes less-informative, resulting in an increased likelihood of less-informative profiles showing high profile similarity [93]. Lineage specific gains and losses of genes, thought to be pervasive in microbial evolution [94], could decrease the similarity between functionally linked genes [74]. Limitations such as these warrant a careful construction of a random null model, preferably on a case-by-case basis, to evaluate the quality of the predicted functional linkages.

## Conclusion

While similarities in phylogenetic profiles of proteins provide helpful clues to functional annotations, there certainly are limitations when it comes to predicting functional linkages on a genomic scale. There are substantial differences in the evolutionary modularity of functional modules [80], which makes it difficult to interpret profile dissimilarity. Recently, several modifications and augmentations to the PPC approach have been proposed in light of its limitations. Jim et al [50] combined organism-to-phenotypic associations with phylogenetic profiles for de novo identification of protein function, and demonstrated the method's superior performance over the basic PPC approach. To detect errors in new functional annotations, Mikkelson et al [55] proposed a probabilistic model of phylogenetic profiles, trained from a database of curated genome annotations. Barker and Pagel [31] and Zhou et al [32] showed that incorporating phylogenetic information (correlated events of gene loss/gain and horizontal gene transfers) into the PPC approach can help distinguish gene pairs that have been gained or lost together on multiple occasions from those that have been gained or lost together once followed by shared inheritance. It was shown that this approach improves the prediction accuracy considerably, and is unaffected by the number of related or redundant genomes in the reference set. However, since this approach relies heavily on the species tree to infer evolutionary events, it may not be suitable in situations when the species tree is unknown or uncertain. To assess the various commonly overlooked fundamental issues related to the performance of PPCs, we undertook a comprehensive analysis of the PPC approach toward functional linkage predictions.

Since not all proteins in an organism date back to the last universal common ancestor, it is important that a large-scale analysis involving protein profile comparisons take into consideration the subsets of proteins showing similar evolutionary behavior, and use reference sets of genomes that take into account the evolutionary histories of individual subsets. This might improve the prediction accuracy given the differential performance of the overall-best reference set BAE3a with respect to particular pathways (Figures 5 and 6). The choice of organisms for the reference set also appears to be an important factor. Sun et al [82] reported that an increased number of genomes in the reference set results in an improved performance. Our results indicate that a carefully chosen set of informative and non-redundant genomes result in a performance as good as if not better than the one obtained from using a larger set of genomes. Simply increasing the number of genomes in the reference set does not improve the prediction accuracy of the PPC approach. In other words, we demonstrated that it is not merely the number of genomes but a careful selection of informative genomes that is essential for a superior performance. In the worst case, as we observed in yeast, adding more eukaryotic genomes only worsens the performance. This result is consistent with Snitkin et al's study [89] that phylogenetic profiling using profiles generated from the current set of completely sequenced eukaryotic organisms yields extremely poor results.

Through a careful case-by-case analysis of proteins from individual KEGG pathways [88], we have shown that there is not one reference set of genomes that can guarantee the best performance in all cases, which suggests that the evolutionary histories of individual pathways or biological systems should be taken into consideration while assembling a set of genomes for phylogenetic profile comparison analysis. This result raises immediate concerns on the genome-wide large-scale functional linkage predictions using profiles constructed from a fixed set of reference genomes despite varying evolutionary histories of individual pathways and network modules.

Hence, based on our observations, we suggest that the following few simple guidelines be followed while selecting genomes for the reference set: 1) Representatives from all 3 kingdoms of life are a must for highly accurate predictions; although reasonably good accuracy can be achieved using prokaryotes alone (please note that this observation is based on extrapolation of our results on *E. coli *and yeast proteins). 2) Naïve collection of genomes should be replaced with a specific choice of genomes representing the entire phylogenetic diversity of life. In particular, fully-sequenced large genomes of free-living organisms give the best information. 3) Parasitic organisms, strains of the same species and very closely related species are unlikely to provide new predictive information, at least for the ensemble of pathways considered in this study. The behavior of phylogenetic profile methods in a general sense is greatly affected by the large-scale evolutionary trends in super-kingdoms. In light of this, it should be kept in mind that the results presented here apply only in the context of the KEGG-derived pathways studied here. One could conceive entirely different rules for inclusion of genomes in the reference set with respect to eukaryote-specific pathways that are not suitably represented in KEGG or were not included in this study. Our observations strongly support the emergence of all extant eukaryotes through a primary endosymbiosis that conferred predominantly bacterial type general metabolism with archaeal type core functional systems. This history is the probable reason for differential performance of PPCs in different functional systems and also the aberrant behavior of eukaryotes in overall analysis.

## Methods

### Data Set and phylogenetic profile construction

The amino acid sequences of 894,522 proteins from 95 different organisms (41 Bacteria, 11 Archaea, and 43 Eukaryotes) were obtained from various sources (NCBI, Ensembl, BROAD, WashU, PlasmoDB, and dictyBase). For comparison of protein sequences against each other, we used NCBI's Basic Local Alignment Search Tool (BLAST) [83]. Every protein *i *is searched against the set of proteins from each organism *j*, and the presence/absence of the query protein's homolog in organism *j *is recorded in the form of BLAST e-value *E*_*ij*_. For each protein *i*, a phylogenetic vector/profile *P *was generated with each entry *P*_*ij *_= -1/log*E*_*ij *_in the vector corresponding to presence/absence information of *i*'s homolog in organism *j*. To avoid logarithm-induced artifacts, values of *P*_*ij *_> 1 are truncated to 1 [64,65,71,72]. Although, earlier works on phylogenetic profile analysis have used binary values (1/0) to record presence/absence of a protein in a given organism [18,32,33,78,84-87], using real values (0.0–1.0), as defined here, provides for different levels of sequence divergence [11,38,72,81].

### Assessing the degree of similarity between two profiles

To assess the similarity between two profiles *A *and *B*, we used the mutual information (MI) measure defined as follows:

MI(*A*, *B*) = *H*(A) + *H*(*B*) - *H*(*A*, *B*),

where H(A)=−∑ap(a)
 MathType@MTEF@5@5@+=feaafiart1ev1aaatCvAUfKttLearuWrP9MDH5MBPbIqV92AaeXatLxBI9gBaebbnrfifHhDYfgasaacH8akY=wiFfYdH8Gipec8Eeeu0xXdbba9frFj0=OqFfea0dXdd9vqai=hGuQ8kuc9pgc9s8qqaq=dirpe0xb9q8qiLsFr0=vr0=vr0dc8meaabaqaciaacaGaaeqabaqabeGadaaakeaacqWGibascqGGOaakcqWGbbqqcqGGPaqkcqGH9aqpcqGHsisldaaeqbqaaiabdchaWjabcIcaOiabdggaHjabcMcaPaWcbaGaemyyaegabeqdcqGHris5aaaa@3A49@ is the entropy of profile *A*, H(A,B)=−∑a,bp(a,b)ln⁡p(a,b)
 MathType@MTEF@5@5@+=feaafiart1ev1aaatCvAUfKttLearuWrP9MDH5MBPbIqV92AaeXatLxBI9gBaebbnrfifHhDYfgasaacH8akY=wiFfYdH8Gipec8Eeeu0xXdbba9frFj0=OqFfea0dXdd9vqai=hGuQ8kuc9pgc9s8qqaq=dirpe0xb9q8qiLsFr0=vr0=vr0dc8meaabaqaciaacaGaaeqabaqabeGadaaakeaacqWGibascqGGOaakcqWGbbqqcqGGSaalcqWGcbGqcqGGPaqkcqGH9aqpcqGHsisldaaeqbqaaiabdchaWjabcIcaOiabdggaHjabcYcaSiabdkgaIjabcMcaPaWcbaGaemyyaeMaeiilaWIaemOyaigabeqdcqGHris5aOGagiiBaWMaeiOBa4MaemiCaaNaeiikaGIaemyyaeMaeiilaWIaemOyaiMaeiykaKcaaa@49F3@ is the joint entropy of profiles *A *and *B*. Here, *p*(*a*) is the frequency with which the value *a *is observed in profile vector *A*, and *p*(*a*, *b*) is the frequency with which the pair of values (a, b) are observed in A and B (with *a *and *b *appearing in *A *and *B*, respectively). Since, our profile entries are real valued numbers in the range 0.0 to 1.0, profile values were binned in intervals of 0.1.

### Pathway similarity calculation

The KEGG pathway database [88] was used to compile the pathway membership information for each protein. The pathway similarity between two proteins *A *and *B*, measuring the degree of their functional linkage, is calculated by taking the Jaccard coefficient of their KEGG database pathway annotation as follows:

Pathway similarity (*A*, *B*) = 100 × (| KEGG_*A *_⋂ KEGG_*B *_|)/(| KEGG_*A *_⋃ KEGG_*B *_|),

where KEGG_x _is the set of specific pathways protein A is known to participate, and |KEGG_x_| is the number of unique pathways in the set. A pathway similarity score of *s *between proteins A and B indicates that A is present in at least *s*% of the pathways that B is present in, and vice-versa.

### Performance measure calculations

To assess the performance of PPCs for prediction of functional linkages, we used the standard measures of sensitivity (coverage), specificity, and positive predictive value (prediction accuracy). Two proteins are considered to be functionally related (positive) if they co-occur in at least one KEGG pathway [42,72], and unrelated (negative) otherwise. For our analysis on individual pathways, we considered a pair of proteins to be a positive if they co-occur in the pathway under consideration. Otherwise, we consider the pair to be a negative (with respect to the pathway under consideration), although they may co-occur in some other pathway. For a chosen mutual information threshold, positives with a score greater than or equal to the threshold are classified as *true positives *and those with a score below the threshold are classified as *false negatives*. Similarly, negatives with score greater than or equal to the threshold are classified as *false positives *and those with a score below the threshold are classified as *true negatives*. The sensitivity measure quantifies the fraction of positives recovered, and is defined as

Sensitivity = TP/(TP+FN),

where TP and FP are the number of true positives and false negatives, respectively, for a particular mutual information threshold. The specificity measure quantifies the fraction of negatives that are correctly identified as negatives, and is defined as

Specificity = TN/(TN+FP)

where TN and FP are the number of true negatives and false positives, respectively, for a particular mutual information threshold. Positive predictive value (PPV) measures the accuracy of predicted functional linkages, and is defined as

PPV = TP/(TP+FP),

where TP and FP are true and false positives for a particular mutual information threshold.

### Statistical significance test

We used *t*-test to determine whether the difference in mean values of two distributions is statistically significant. Given two distributions *P *and *Q*, each characterized by its mean, standard deviation, and the number of data points, the *t*-test measures whether the means are distinct. First, the *t*-score, which measures the signal (difference between two means) to noise (variability in distribution) ratio, is calculated as follows:

t=P¯−Q¯var⁡PnP+var⁡QnQ,
 MathType@MTEF@5@5@+=feaafiart1ev1aaatCvAUfKttLearuWrP9MDH5MBPbIqV92AaeXatLxBI9gBaebbnrfifHhDYfgasaacH8akY=wiFfYdH8Gipec8Eeeu0xXdbba9frFj0=OqFfea0dXdd9vqai=hGuQ8kuc9pgc9s8qqaq=dirpe0xb9q8qiLsFr0=vr0=vr0dc8meaabaqaciaacaGaaeqabaqabeGadaaakeaacqWG0baDcqGH9aqpdaWcaaqaaiqbdcfaqzaaraGaeyOeI0IafmyuaeLbaebaaeaadaGcaaqaamaalaaabaGagiODayNaeiyyaeMaeiOCai3aaSbaaSqaaiabdcfaqbqabaaakeaacqWGUbGBdaWgaaWcbaGaemiuaafabeaaaaGccqGHRaWkdaWcaaqaaiGbcAha2jabcggaHjabckhaYnaaBaaaleaacqWGrbquaeqaaaGcbaGaemOBa42aaSbaaSqaaiabdgfarbqabaaaaaqabaaaaOGaeiilaWcaaa@4538@

where P¯
 MathType@MTEF@5@5@+=feaafiart1ev1aaatCvAUfKttLearuWrP9MDH5MBPbIqV92AaeXatLxBI9gBaebbnrfifHhDYfgasaacH8akY=wiFfYdH8Gipec8Eeeu0xXdbba9frFj0=OqFfea0dXdd9vqai=hGuQ8kuc9pgc9s8qqaq=dirpe0xb9q8qiLsFr0=vr0=vr0dc8meaabaqaciaacaGaaeqabaqabeGadaaakeaacuWGqbaugaqeaaaa@2DED@ and var_*P *_are the mean and the variance of *P*, respectively, and *n*_*P *_is the number of data points in *P*. The significance of computed *t*-score is tested by comparing it against the value for risk level 0.001 (odds that the differences in mean is due to chance) and degrees of freedom (*n*_*P*_+*n*_*P*_-2) from a standard table of significance to determine whether the computed *t*-score is large enough to be significant. If it is, then the difference in two means is statistically significant. For sizes of datasets in our study, the computed *t*-score should be greater than 3.29 (for risk level 0.001) in order to reach a conclusion that the observed difference in means of the two distributions is statistically significant. The risk level of 0.001 indicates that that one out of a thousand times, you would find a statistically significant difference between the means even if there was none (i.e., by "chance"). The higher the *t*-score, the higher the significance.

## List of abbreviations

PPC – Phylogenetic Profile Comparison

## Authors' contributions

RJ conceived, designed and implemented the study. RJ and LA analyzed and interpreted the results, and drafted the manuscript. TMP participated in the project design, and revision of the manuscript.

## Supplementary Material

Additional file 1 Set of 155 bacterial-specific *E. coli *proteins. Phylogenetic profiles of 155 *E. coli *proteins, none of which had a homolog in any of the archaeal and eukaryotic genomes (e-value > 1e-2) used in this study. Red and blue colors indicate the presence (lower values in the profile) or absence (higher values in the profile) of a homolog, respectively. For example BLAST e-values 1e-10, 1e-5, 1e-3, 1e-2 and 1e-1 are represented as 0.03, 0.06, 0.10, 0.15, and 0.30, respectively, in profiles (see Materials and Methods). Click here for file
