# Supplementary figures and images for: Discovering functional linkages and uncharacterized cellular pathways using phylogenetic profile comparisons: a comprehensive assessment
Source: BMC Bioinformatics. 2007 May 23;8:173. doi: 10.1186/1471-2105-8-173 (PMC1904249; doi:10.1186/1471-2105-8-173)

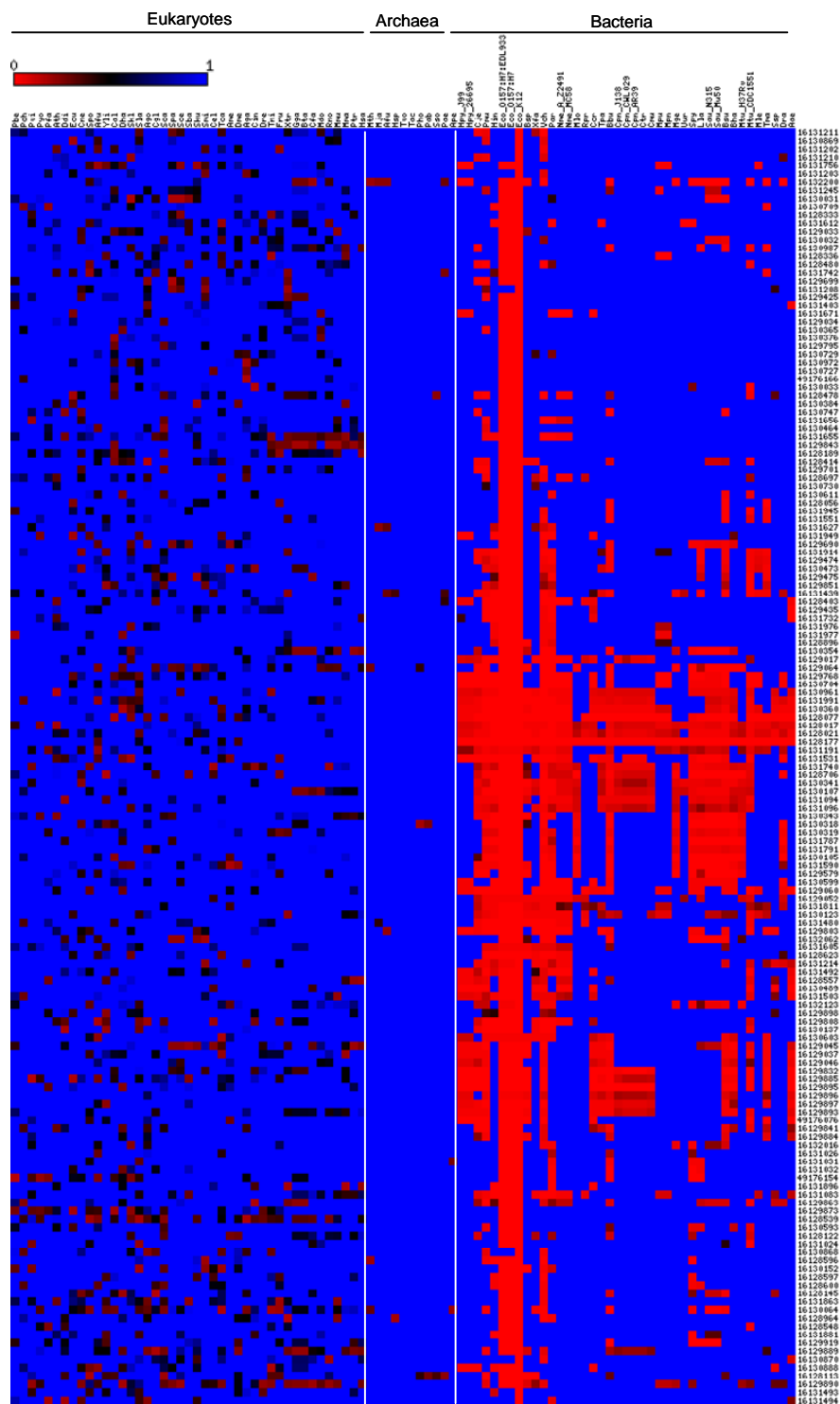

Supplement: Additional file 1 — Set of 155 bacterial-specific E. coli proteins. Phylogenetic profiles of 155 E. coli proteins, none of which had a homolog in any of the archaeal and eukaryotic genomes (e-value > 1e-2) used in this study. Red and blue colors indicate the presence (lower values in the profile) or absence (higher values in the profile) of a homolog, respectively. For example BLAST e-values 1e-10, 1e-5, 1e-3, 1e-2 and 1e-1 are represented as 0.03, 0.06, 0.10, 0.15, and 0.30, respectively, in profiles (see Materials and Methods). [file 1471-2105-8-173-S1.pdf]
